# Probiotics, gut microbiota, and brain health: Exploring therapeutic pathways

**DOI:** 10.3934/microbiol.2025022

**Published:** 2025-07-08

**Authors:** Srirengaraj Vijayaram, Karthikeyan Mahendran, Hary Razafindralambo, Einar Ringø, Suruli Kannan, Yun-Zhang Sun

**Affiliations:** 1 Xiamen Key Laboratory for Feed Quality Testing and Safety Evaluation, Fisheries College, Jimei University, Xiamen 361021, China; 2 Centre for Global Health Research, Saveetha Medical College and Hospital, Saveetha Institute of Medical and Technical Sciences, Saveetha University, Thandalam, Chennai-602 105, Tamil Nadu, India; 3 Department of Microbiology, PSG College of Arts and Science, Coimbatore, Tamil Nadu, India; 4 ProBioLab, B-5004 Namur, Belgium; 5 BioEcoAgro Joint Research Unit, TERRA Teaching and Research Centre, Microbial Processes and Interactions, Gembloux Agro-Bio Tech/Université de Liège, B-5030 Gembloux, Belgium; 6 Norwegian College of Fishery Science, Faculty of Bioscience, Fisheries, and Economics, UiT the Arctic University of Norway, Tromsø, 9037, Norway; 7 Department of Environmental Studies, School of Energy, Environment and Natural Resources, Madurai Kamaraj University, Madurai, India

**Keywords:** probiotics, microbiome, gut–brain axis, gut microbiota, neurological health, microbiota-gut–brain interaction, neurodegenerative disorders

## Abstract

The gut microbiome plays a significant role in regulating gastrointestinal (GI) function and modulating the gut–brain axis, which describes the bidirectional communication between the GI tract and the central nervous system (CNS). Its involvement in digestion, immunity, and neurophysiology is well recognized. This study offers novel insights by focusing on psychobiotics, a class of probiotics with targeted neuroactive properties. These microorganisms influence brain function through defined mechanisms, including modulation of neuroinflammation, neurotransmitter production (GABA, serotonin), regulation of the hypothalamic–pituitary–adrenal (HPA) axis, and vagus nerve signaling. Our work critically examines recent advances in applications of psychobiotics for neurological disorders such as Parkinson's disease, Alzheimer's disease, multiple sclerosis, and autism spectrum disorder. By integrating evidence from microbiome research, neuroimmunology, and clinical studies, we identify promising microbial strains and mechanistic pathways with therapeutic potential. This study contributes original perspectives by highlighting underexplored microbe–host interactions and proposing targeted microbial interventions as adjuncts to conventional neurotherapies. Further research is needed to validate strain-specific effects, long-term efficacy, and safety profiles in clinical settings.

## 1. Introduction

The concept of the gut–brain interaction dates to the 1880s and was articulated by William James and Carl Lange. This idea postulates that there is a two-way movement of signals between the gut and the brain. Signals from the brain can impact numerous features of gastrointestinal (GI) function, including motor function, sensory perception, and secretory processes. Conversely, signals originating in the gut can affect emotional responses, stress levels, and the body's modulation of pain perception. In the past 15 years, there has been a flood of research focusing on the role of intestinal microbiota in affecting the brain through the gut–brain interaction. This study has revealed the significant role that gut microbiota plays in the body's response to stress. Particularly, studies have shown that in germ-free mice (mice lacking a gut microbiota), there is an increase in the production of stress markers such as plasma ACTH (adrenocorticotropic hormone) and corticosterone, whereas specific pathogen-free mice have a well-established gut microbiota. This effect is particularly observed under conditions of controlled stress [1].

During primary growth, the gut microbiota plays a key role in shaping the growth and functioning of the central nervous system (CNS). The development of microbial colonization begins shortly after birth and is authoritative in recruiting signaling responses that impact various aspects of the CNS's development. As the gut microbiota colonizes the gastrointestinal (GI) tract, it interacts with the host's body and the gut–brain axis. This communication leads to the recruitment of signaling responses that have far-reaching effects on neuronal circuits in the CNS. The signaling responses triggered by the gut microbiota have been shown to influence the neuronal circuits involved in various functions, including motor control and emotional regulation. These routes are multifaceted and can impact an individual's ability to control movement and their responses to stress, anxiety, and other emotional states [2]. The CNS and enteric nervous system (ENS) constitute a bidirectional communication system known as the gut–brain axis. This system connects various emotional and cognitive areas of the brain with the outermost functions of the GI tract [3].

The gut microbiota in healthy individuals comprises a diverse community of microorganisms, primarily bacteria. This microbial community, often called the gut microbiome, plays several important roles in maintaining human health [4]. The human GI tract is home to a vast number of microorganisms. It is estimated that there are approximately 10^13^ to 10^14^ bacteria in the human gut. This population of bacteria is so dense that it can collectively weigh around 1–2 kilograms [5]. It is important to clarify that there is a wide variety of bacterial phyla on Earth, but not all colonize the mammalian intestine. Most of Earth's bacterial phyla do not have the slightest association with the human or mammalian gut. Phyla like Firmicutes, Bacteroidetes, Actinobacteria, and Proteobacteria are commonly found in the human gut microbiome [6]. The gut microbiota maintains the host's health, and microbial metabolites are integral to this interaction. The gut microbiota contributes to the host's health through various mechanisms, including beneficial microbial metabolites [7]. The gut microbial community has a multifaceted and dynamic relationship with humans that profoundly influences many aspects of health and well-being [8]. The gut microbiota plays a vital role in maintaining the host's health and the proper functioning of the intestines [9],[10]. The gut microbiota plays a crucial role in bile acid metabolism and various beneficial metabolites that can impact human health [11].

Dietary components such as inulin and fructooligosaccharides (FOS) can stimulate the gut microbiota's composition and lead to changes in the growth of specific bacterial species, including *Lactobacillus* species and *Bifidobacteria*. The effect of fructan-supplemented diets on the gut microbiota aligns with previous research. Fructans, including inulin and FOS, are types of prebiotic fibers known to affect the composition and activity of the gut microbiota [12]. The gut microbiota contributes to the immune system's balance and completes the production of short-chain fatty acids (SCFAs). The interaction between SCFAs and immune cells is a complex and finely tuned process that helps maintain gut homeostasis [13]–[15]. The ability of SCFAs to modulate the immune system through epigenetic mechanisms like histone alterations highlights the intricate interplay among the gut microbiota, dietary factors, and the host immune system. Understanding these mechanisms may provide insights into developing treatments for autoimmune diseases, allergies, and other conditions related to immune dysfunction [16],[17]. The gut–brain axis is a complex and bidirectional communication system involving various mechanisms, including the influence of gut microbial metabolites such as SCFAs on gut and brain function. Understanding these interactions is crucial for unravelling the connections among gut health, immune regulation, and neurological well-being [18]. Given the importance of the gut–brain axis, the gut microbiome's imbalance in the etiology and symptoms of neurological disorders may be crucial for maintaining brain health. As an alternative therapy for neurological disorders, probiotics may serve as safe agents that positively affect the gut microbiome's balance [19]. Trillions of microorganisms exist in the human gut, interacting with the host cells to influence and support bodily processes. Research investigating variables that could impact the gut microbiome and be applied therapeutically for disorders such as dysbiosis is particularly intriguing [20]. The neurological, immunological, and chemical signal networks comprising the brain–gut–microbiota axis form part of a complex and dynamic system. It appears that the pathophysiology of neurological disorders involves all these networks. The vagus nerve, microglia, gut microbial composition, Th17/Treg activity, and SCFAs have garnered increased attention in efforts to understand the brain–gut–microbiota axis's role. This axis is viewed as a novel paradigm that may offer new approaches to treating these disorders [21]. Dalton, et al. [22] conducted an interesting study exploring how exercise influences the microbiome–gut–brain axis. The complex communication pathway, i.e., the microbiome–gut–brain axis, reflects the surrounding multicellular organism. The microecosystem is symbiotic, and the diversity and balance of bacterial phyla support overall health. Declines in gut microbial diversity can lead to systemic effects, such as gastrointestinal and psychological discomfort. Treatments that influence the microbiome, such as probiotic supplements, have been initiated to recover gut and neurological disorders [22]. This study employs a critical, evidence-based synthesis of recent primary research, including clinical trials and mechanistic studies, to evaluate the therapeutic role of psychobiotics in modulating the gut–brain axis. Rather than merely summarizing the existing literature, it categorizes and analyzes data according to the probiotic strains, neurological outcomes, and mechanisms of action. Emphasis was placed on high-quality, peer-reviewed sources, enabling a focused assessment of efficacy and the identification of gaps for future research. This approach ensures methodological rigor and contributes original insights into the clinical potential of psychobiotics.

## 2. Neurological factors

Gut microbial diversity plays a significant role in various age-related neurological disorders and can be influenced by a range of influences, inherent and extrinsic [23]. The gut microbiota plays a vital role in producing various neuromodulator substances that can affect the host's neuronal functions locally in the gut and centrally in the brain [24]. The gut microbiota can synthesize different kinds of neuroactive elements like serotonin, tryptamine, tryptophan, dopamine, oxytocin, gamma-aminobutyric acid (GABA), acetylcholine, and noradrenaline, which can influence the functioning of the nervous system and have far-reaching effects on the body. These neuroactive elements show the extensive communication between the gut microbiota and the nervous system, forming the basis of the gut–brain axis. The gut–brain axis involves a bidirectional relationship, with the gut microbiota inducing neurological function and, in turn, the nervous system affecting gut health and function. Moreover, the production of these neuroactive elements by the gut microbiota underscores the significance of maintaining a balanced and diverse gut microbial community for overall well-being, including emotional and cognitive health [25].

The gut microbiota synthesizes a wide range of neurotransmitters and neuromodulators, and these microbial components have an impact on gut–brain communication and functions. This intricate network of interactions between the gut and the brain is often referred to as the gut–brain axis [26]–[28]. *Lactobacillus* species are commonly reported in the gut microbiota and have been shown to affect the concentration and expression of GABA receptors in the CNS, primarily through the gut–brain axis. GABA, or gamma-aminobutyric acid, is a major inhibitory neurotransmitter in the brain, and its receptors play a crucial role in regulating brain function and mood [29],[30]. Gamma-aminobutyric acid (GABA) is a major inhibitory neurotransmitter in the nervous system, and the gut microbiota, including *Bifidobacteria* and *Lactobacillus*, can produce GABA. It is important to note that the quantity of GABA produced by the gut bacteria is relatively low compared with the overall GABA levels in the CNS. However, even this relatively low quantity can have significant effects on the gut–brain axis [31],[32]. The synthesis of 5-hydroxytryptamine (5-HT), commonly known as serotonin, occurs predominantly in the gut and is essential for gut motility and signaling. The gut microbiota plays a significant role in regulating the synthesis of serotonin, particularly through interactions with enterochromaffin cells (ECs) in the colon [33].

Probiotics, particularly strains of beneficial bacteria, have been shown to stimulate the regulation of serotonin (5-HT) and the expression of the serotonin transporter (5-HTT) in the gut–brain axis [34]. The expression of neuropeptides in primary sensory neurons, namely neuropeptides and calcitonin gene-related peptide (CGRP), is significant attention in the situation of intestinal pain and sensory processing. The exact mechanisms of how the gut microbiota influences the expression of these neuropeptides in primary sensory neurons are still under investigation [35]. The GI tract is recognized as the body's largest endocrine organ, and it plays an important role in synthesizing different kinds of bioactive peptides and hormones that have far-reaching effects on multiple physiological processes [36]. Various peptides synthesized and released by the gut play important roles in regulating different types of physiological and behavioral processes [37]–[39]. Neuropeptides like galanin play a role in regulating the body's response to stress and can influence the function of the hypothalamic–pituitary–adrenal (HPA) axis. The HPA axis is a critical component of the body's stress response system. When an individual encounters a stressor, whether physical or psychological, the HPA axis is activated, leading to the release of hormones such as corticotropin-releasing hormone (CRH), adrenocorticotropic hormone (ACTH), and cortisol [40],[41]. Microbe-associated molecular patterns (MAMPs) are components of microorganisms, such as bacteria, that are recognized by the pattern recognition receptors (PRRs) of the host's innate immune system. These interactions are essential for the host's defense against potential microbial threats. Two significant MAMPs are lipopolysaccharide (LPS) and peptidoglycan, specifically meso-diaminopimelic acid, and these interactions play a vital role in the host's ability to recognize and respond to potentially harmful bacteria. By detecting these MAMPs, the innate immune system can initiate defense mechanisms, including the recruitment and activation of immune cells, to fight off infections and maintain overall health. The gut immune system plays a significant role in this process as it acts as a frontline defense against pathogens that are encountered through the digestive system [42].

## 3. Immune system response

The key findings from previous studies show that GF mice exhibit a range of immune disorders, with abnormal numbers of several immune cell types and altered cytokine profiles. The gut microbiota community is vital for the proper development and function of the immune system. The absence of the gut microbiota community in GF mice resulted in deficits in local and systemic lymphoid structures that are essential components of the immune system. GF animals displayed significant differences in various ENS and CNS diseases. These differences included susceptibility to neurodegenerative disorders, anxiety, and depression. The expression of the brain-derived neurotrophic factor (BDNF) gene in areas of the brain, such as the amygdala and hippocampus, was reduced in GF mice. BDNF is a critical factor in the growth, survival, and maintenance of neurons, and its alteration can affect brain function and mental health. GF mice showed increased permeability of the blood–brain barrier (BBB). A compromised BBB can allow the passage of substances and immune cells from the bloodstream into the brain, potentially contributing to neurological disorders. The HPA axis plays a central role in the body's response to stress and is hyperactivated in GF mice. This hyperactivation can have wide-ranging effects on physiological and psychological health. Microglia, the resident immune cells of the CNS, showed signs of dysfunction in GF mice. Proper microglial function is critical for brain health and immune regulation within the CNS [43],[44]. The interaction of the immune system, the nervous system, and the gut microbiota represents a complex and dynamic process that influences human health, starting from early life stages, and continues to play an important role throughout the lifespan. Understanding this interplay is vital for endorsing and maintaining health, particularly during early development [45],[46]. The immune system helps as a pivotal intermediary in the dynamic equilibrium that exists between the brain and the gut. This intricate and bidirectional interaction is often referred to as the gut–brain axis (GBA). The GBA functions as a communication network, facilitating the exchange of information between the CNS and the ENS. Importantly, the immune system acts as a main mediator in this communication. Within the gut, the immune system senses and responds to potential threats, including pathogens and foreign substances. Immune activation involves the delivery of cytokines, which are small proteins that mediate and control immunity, inflammation, and hematopoiesis. In the context of the GBA, cytokines such as interleukin-6 (IL-6), interleukin-1β (IL-1β), and tumor necrosis factor-alpha (TNF-α) can cross the blood–brain barrier (BBB) or act on the vagus nerve, influencing brain function, behavior, and mood. Cytokines produced in response to gut microbiota signals or dysbiosis can initiate or exacerbate neuroinflammation. A central component of this process involves the microglial cells, the resident immune cells of the CNS. Microglia become activated upon sensing systemic inflammatory signals and microbial components such as lipopolysaccharides (LPSs). Once activated, the microglia release proinflammatory mediators, reactive oxygen species (ROS), and additional cytokines, potentially disrupting synaptic function and neuronal signaling, and contributing to the pathogenesis of neuropsychiatric and neurodegenerative disorders. Moreover, the gut hosts a diverse community of microorganisms, collectively known as the gut microbiota, that interact closely with the immune system. These microbes can help to regulate the balance between proinflammatory and anti-inflammatory responses. The gut-associated lymphoid tissue (GALT) plays an imperative role in orchestrating innate and adaptive immune responses, maintaining tolerance to beneficial microbes, and mounting defenses against pathogens [47]. Modulation of significant cytokines such as TNF-α by probiotics has been shown to yield either immunostimulatory or immunosuppressive effects, depending on the host's immune status and microbial context [48],[49]. In this way, gut microbes can modulate, tune, and refine the host's immune responses, ultimately inducing the state of the gut–brain axis [50]. Oral tolerance is a physiological mechanism where the immune system learns to tolerate and not mount an immune response against harmless antigens, such as food proteins, to prevent unnecessary inflammation. This is important for sustaining a balanced immune response in the gut, which is constantly exposed to a wide range of antigens from the diet. In the process of oral tolerance, there is a shift towards a Th2 response and a downregulation of the Th1 response. This process helps reduce inflammation and immune reactions to dietary antigens, promoting immune tolerance in the gut. This delicate balance is crucial for preventing hypersensitivity reactions, allergies, and autoimmune responses to dietary antigens. It is also a significant aspect of maintaining gut health and overall immune homeostasis. Understanding these mechanisms is important for addressing immune-related disorders and designing therapeutic approaches to modulate immune responses in the gut and elsewhere in the body [51].

The gut microbiota plays a pivotal role in educating the immune system to recognize foreign antigens, promoting tolerance toward commensal microbes. This educational process is vital for maintaining immune homeostasis and overall health [52]. In some cases, individuals may develop autoantibodies against specific neuropeptides and hormones involved in appetite control. Autoantibodies are wrongly target and attack the body's proteins or molecules. These autoantibodies target neuropeptides and hormones linked to appetite regulation, and they can have been suggested to affect individuals' appetite and metabolic processes [53]–[55]. Recent research has unveiled specific properties of the colonic microbiota alongside the local gut immune system and the distal (systemic) immune system, with significant implications for overall health and disease [56]. Dysbiosis, an imbalance or disruption in the composition and function of the gut microbiota, has been associated with various health issues, including the development of autoimmunity. The autoimmune system mistakenly targets and attacks the body's tissues and cells [57]. The gut microbiota may play a role in developing or exacerbating autoimmune encephalomyelitis. Changes in the gut microbiota's composition and function can potentially influence the immune responses associated with this condition [58] and the potential connection between the gut microbiota and autoimmune demyelination. Changes in the gut microbiota may trigger or contribute to the autoimmune responses observed in these conditions [59]. The term “autoimmune encephalomyelitis” typically refers to a group of experimental autoimmune diseases, with the most well-known being experimental autoimmune encephalomyelitis (EAE). EAE is used as an experimental model to study autoimmune demyelinating diseases of the CNS, particularly multiple sclerosis (MS). It is characterized by inflammation and demyelination of the CNS, leading to neurological symptoms [53]–[55]. Treatment of rodents with the probiotic *Limosilactobacillus reuteri* has been shown to attenuate sensory neuron hypersensitivity, suggesting an interesting connection between modulation of the gut microbiota and the function of sensory neurons [60]. The observation that treatment with *L. reuteri* alleviates the pain-related response to gastric distension indicates a potential role for this probiotic in managing conditions characterized by abdominal discomfort or pain, especially in response to gastric (stomach) distension [61]. The gut–brain axis is a complex bidirectional communication system that plays a crucial role in various physiological processes, and suggests that factors like diet, stress, and the gut microbiota can have a noteworthy effect on gut health and mental health. This field of research continues to expand, revealing new insights into how the gut and brain influence each other and overall health [62]. The microbiota–gut–brain axis is an extremely dynamic and multifaceted network of interactions. Ongoing research aims to unravel the precise mechanisms through which gut microbes stimulate brain health, cognitive function, and behavior. This area of study has significant implications for understanding and potentially treating a range of neurological and mental health conditions. It highlights the interconnectedness of the gut, immune system, endocrine system, and brain, emphasizing the importance of a healthy gut microbiota for overall well-being [63].

## 4. Gut–brain axis cell signaling pathways

**Figure 1.**
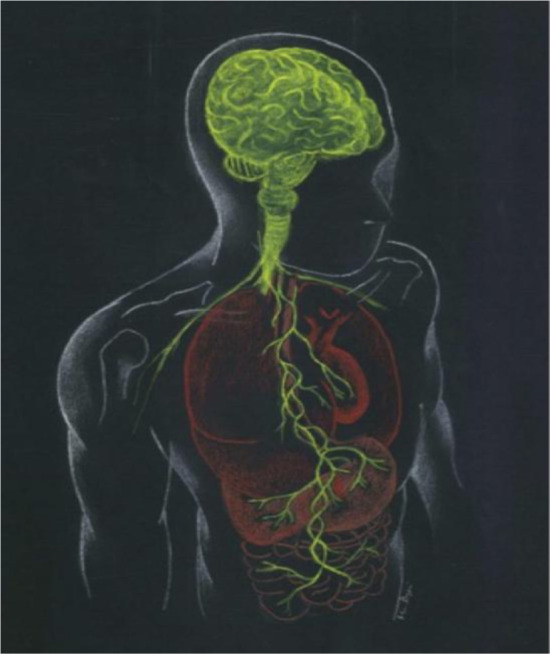
Schematic representation of the microbiota–gut–brain axis, illustrating the complex bidirectional communication of the gut microbiota, the ENS, and the CNS. Signals are transmitted through neural (vagus and sympathetic nerves), endocrine (hormones), and immune (cytokines) pathways [64].

The term “GBA” or gut–brain axis is commonly used to refer to the bidirectional communication system that associates the GI tract with the CNS, including the brain. This term has been extended to the microbiota–gut–brain axis, since the gut microbiota is of primary importance to this pathway (Figure 1) [64]. It indicates the dynamic and intricate relationship between the gut and the brain. Recently, there has been a growing recognition of the role of the intestinal microbiota in inducing the functioning of the gut–brain axis [65].

The inflammasome is a critical component of the innate immune system that responds to various danger signals and microbial pathogens. It triggers an inflammatory response by activating caspase-1, which, in turn, processes pro-IL-1β and pro-IL-18 into their active forms. This process is an essential part of the body's defense against infections and cellular stress [66]. Type I interferon (IFN-I) is a versatile cytokine that is induced in response to pathogen-associated molecular patterns (PAMPs) and is essential for priming the host's immune responses. It acts through a network of pattern recognition receptors (PRRs), including Toll-like receptors (TLRs), the nucleotide-binding domain and leucine-rich repeat-containing gene family, and RIG-I-like receptors. IFN-I plays a critical role in the immune system's capability to respond to viral, bacterial, and tumor components, contributing to the maintenance of host homeostasis and protection against infections and disease [67],[68]. TLRs activate the NF-κB pathway through the recruitment of the adaptor molecule MyD88, which is a critical step in the beginning of the innate immune response to pathogens [69]. The NF-κB family of transcription factors is a central player in the immune system. It bridges the gap between innate and adaptive immune responses, influences immune cell function, and contributes to the maintenance of the immune system's balance. Its role is vital in the host's defense, immune regulation, and immune-related disorders [70]. The gut microbiota has been implicated in playing a crucial role in a bidirectional communication pathway known as the gut–brain axis. This pathway enables the gut microbiota to communicate not only with local GI cells (epithelial, immune, and nerve cells) but also with distant organs, including the brain. The gut microbiota generates and releases various molecules, pathogen-associated molecular patterns (PAMPs), or microbe-associated molecular patterns (MAMPs). These molecules can act as signals to affect the function of different organs and systems in the body. In addition to PAMPs and MAMPs, the gut microbiota produces a wide range of microbial metabolites. These metabolites include products of microbial metabolism and fermentation. Many of these metabolites are bioactive and can have systemic effects. Experimental evidence suggests that a significant portion of the metabolites present in the bloodstream of mammals are derived from the metabolic activities of the intestinal microbial community. These microbial metabolites can be transported via the circulatory system to different organs and tissues, disturbing their function and contributing to various physiological processes [71]–[74]. Metabolic byproducts produced by the gut microbiota, including short-chain fatty acids (SCFAs) and gases like CO_2_ (carbon dioxide), CO (carbon monoxide), H_2_S (hydrogen sulfide), and NH_3_ (ammonia) play crucial roles in human health and can have significant impacts on different characteristics of the body's physiology and disease processes [75],[76],[10]. The gut can communicate with the brain through hormonal signaling pathways that involve the release of gut peptides from enteroendocrine cells. These gut peptides can directly affect the brain and play a central role in regulating various physiological processes [77]. The gut microbiota is a bidirectional communication system acting via neural, immune, and endocrine pathways [78],[79]. The gut microbiota stimulates the growth and functions of the ENS through some mechanisms, including the triggering of pattern recognition receptors, specifically Toll-like receptor (TLR)-2 and TLR4 [80]. The activation of Toll-like receptors (TLRs) can be finely modulated through their interactions with sialic acid-binding immunoglobulin-like lectins (Siglecs). Siglecs are recognized for their inhibitory effects on immune responses. The extensive interactions between Siglecs and TLRs serve to downregulate and attenuate the activation of TLRs, exerting a negative regulatory influence on the immune response [81]. Elevated ammonia levels in the brain lead to the binding of ammonia with glutamate, catalyzed by glutamine synthetase, resulting in the formation of glutamine. This accumulation of glutamine and ammonia is a known factor associated with brain edema. Research has indicated that the abnormal uptake, transport, and delivery of glutamate contribute to an increase in extracellular glutamate concentration. Additionally, irregular functioning of the glutamate–nitric oxide–cyclic guanosine monophosphate (NO-cGMP) metabolic pathway in the brain is linked to impairments in learning and memory [82],[83]. Gut-to-brain signaling involves highly chemosensitive primary afferent neurons, immune cells, and enteroendocrine cells that secrete more than 30 different hormones. Additionally, immune pathways, such as cytokine signaling triggered by microbial lipopolysaccharide (LPS) or peptidoglycan, serve as crucial communication links to the brain. Transformed gut barrier integrity can potentially lead to the translocation of these microbial products into the periphery, subsequently causing microglial activation and neuroinflammation [84]. Cell signaling pathways are responsible for stimulating TNF-α production in the human body and involve multiple molecules and alternative routes. These pathways can be triggered by substances present in the cell walls of various microorganisms, including LPSs from Gram-negative bacteria, and lipoteichoic acid (LTA) and peptidoglycans from Gram-positive bacteria. Stimulation of these pathways can result in a systemic inflammatory response [85]. Signal transduction is a complex process that can incorporate neural, endocrine, immune, and metabolic pathways. However, the detailed mechanisms and signals complicated in this process still require clarification [86]–[88]. Recent studies have explored the intricate interplay between gut–brain microbiota and its impact on health and disease. The GI system houses an ENS, akin to a neuronal network within the spinal cord. These networks enable brain signals to control the gut's secretory and sensory functions. Communication between the brain and gut microbes happens through numerous physiological channels, including neuroendocrine pathways, the autonomic nervous system, neuroimmune pathways, and signaling molecules produced by the gut microbes [89]–[93]. In the gut, favoring the modulation of the kynurenine pathway towards increased kynurenic acid synthesis may serve as a neuroprotective mechanism to counteract the elevated NMDA receptor activity induced by inflammation in the neuromuscular compartment [43]. Enteric NMDA receptor pathways could potentially support the development of neuroinflammatory responses by triggering the activation of oxidative and nitrosative stress pathways [94]. Butyrate, the main energy source for human colonocytes, plays a complex role in maintaining colonic health. It protects colon cells against cancer by activating apoptotic cascade pathways, supports glucose and energy homeostasis, triggers beta-oxidation in epithelial cells to restore gut oxygen balance, and mitigates gut microbiota dysbiosis. Moreover, transcriptomic analysis of the microbiome has shown that butyrate enhances the expression of genes associated with fatty acid oxidation, the electron transport chain, and oxidative stress pathways [95]. Butyric acid is paramount in nourishing these cells, simultaneously serving as a key stimulant for their growth and differentiation [96]. Propionate interacts with fatty acid receptors, influencing satiety and liver gluconeogenesis. Meanwhile, acetate plays a key role in promoting gut microbial growth, regulating cholesterol metabolism and peripheral tissue lipogenesis [97]. The metabolism of tryptophan (Trp) is driven by three major pathways, namely the kynurenine pathway, the direct pathway, and the serotonin pathway. The kynurenine pathway metabolizes about 90% of Trp into various metabolites, including kynurenic acid, xanthurenic acid, picolinic acid, quinolinic acid, and nicotinamide adenine dinucleotide (NAD). The kynurenine pathway is considered crucial because several neuropsychiatric disorders have been reported to involve the dysfunction of specific steps in this pathway due to acquired or inherited enzyme deficiencies [98]. Trp is an essential amino acid, meaning that it cannot be synthesized within the human body and must be obtained through dietary sources. Rich dietary sources of Trp include chocolate, eggs, fish, dairy products, legumes, and meat [99],[100].

## 5. The effect of gut microbes on gut health and brain functions

**Figure 2.**
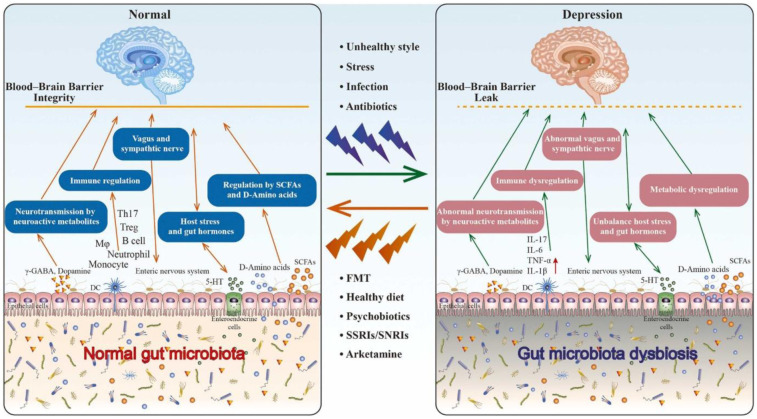
Mechanistic insights into the gut–brain–microbiota axis in depression. The left panel shows a healthy state with the intact gut microbiota, blood–brain barrier (BBB), and neurotransmission. The right panel illustrates dysbiosis-induced alterations, including BBB leakage, immune dysregulation, and abnormal neuroendocrine signaling. These changes contribute to the onset and progression of depressive symptoms. Therapeutic strategies such as psychobiotics, a healthy diet, and fecal microbiota transplantation (FMT) are indicated to restore balance [21].

The role of the gut microbiota in depression was studied through an analysis of the gut–brain axis by Irum, et al. [101]. An unhealthy lifestyle due to increased and sustained stress, infection, or other factors can cause gut microbiota dysbiosis. The brain may bidirectionally control irregular conditions in the body–gut–microbiota axis via neural signals, immune signals, or chemical signals, thereby contributing to depression. On the contrary, Suganya and Koo [102], in their study, mentioned that the gut microbiota has the possibility to improve brain function, as well as the progression of neurological disorders. Those authors also described how the gut microbiota can produce the neurotransmitter dopamine (3,4-dihydroxyphenylacetic acid, DOPAC) in depressive persons. The role of the brain–gut–microbiota axis in depression is shown in Figure 2.

## 6. Neurological disorders and diseases

Neurodegenerative diseases affect millions of individuals worldwide, and the global improvements in health, driven by clinical research, have improved potential human lifespan and the economic burden of these diseases [103]. According to the World Health Organization's predictions, by 2040, neurodegenerative diseases are predicted to surpass cancer as the second leading global cause of death [104]. Neurodevelopmental disorders (NDDs) encompass a range of heterogeneous syndromes characterized by functional impairments in the CNS due to disturbances in essential neurodevelopmental processes [105]. In addition, lifestyle plays a vital role, as studies have specified that individuals with sedentary cognitive and physical habits are at a higher risk of developing mild cognitive impairment (MCI) [106]. Additionally, dietary patterns, antibiotic usage, and bacterial and viral infections are often connected to changes in gut bacterial structure and interruptions in gut homeostasis. These factors have been implicated in developing gut–brain disorders [107]. Neurodegenerative diseases like Parkinson's disease (PD) and Alzheimer's disease (AD) include a diverse group of disorders that lead to the degeneration of the central and/or peripheral nervous systems. PD affects around 1% of the population; AD influences approximately 8% [108]. The composition of the microbiota differs significantly between healthy individuals and those with neurodegenerative disorders, including multiple sclerosis (MS), AD), and PD [109]–[114], and neuropsychiatric disorders (NPSs) [115]. Conditions like major depressive disorder and mood disorders are increasingly related to the bidirectional communication between the gut and the brain. This communication is vital for maintaining brain function and gut homeostasis. Neurological disorders, including PD, AD, MS, autism spectrum disorder (ASD), and stress, are known to alter this bidirectional relationship, leading to the development of brain–gut disorders like irritable bowel syndrome (IBS) and others [116],[117]. IBS is characterized by a disrupted gut–brain axis homeostasis and is linked to symptoms like abdominal visceral pain and changes in bowel habits. It often co-occurs with anxiety and depression. Researchers have utilized animal models of visceral hypersensitivity to investigate the role of the gut microbiota in visceral pain pathways [118],[119]. Stress, in combination with diet, may play a role in the development of IBS. Multiple studies comparing IBS patients with normal controls have shown reduced levels of *Lactobacillus* and *Bifidobacterium* genera, along with elevated Firmicutes to Bacteroidetes ratios at the phylum level [120],[121]. At the molecular level, neuroinflammation, oxidative stress, and excitotoxicity are closely connected to numerous neurological disorders. Accumulated oxidative stress is considered a key mechanism contributing to cognitive impairment and neurodegenerative diseases, such as AD [122]. Cognitive functions are the most intricate capacities of the nervous system, responsible for rational perception, cognition, and interaction with the external environment. They play a central role in performing complex, intellectual tasks and everyday household actions [123]. The stress response is intricately regulated by interconnected brain regions, including the amygdala, hippocampus, and the paraventricular nucleus of the hypothalamus. These regions also receive modulatory inputs from higher cortical areas, like the prefrontal cortex [124],[125]. Depression and general anxiety disorders have well-established links to traumatic life events, especially experienced early in life or during periods of chronic stress in humans and animals [126]–[128].

### 6.1. Autism spectrum disorder

The term “autism spectrum disorder” (ASD) encompasses a group of diverse neurodevelopmental disorders with various causes and courses, variable symptom severity, and numerous associated comorbid conditions, including anxiety and GI symptoms [129]. The fifth edition of the *Diagnostic and Statistical Manual of Mental Disorders* (DSM-5) defines autism as a condition marked by specific features [130]. The causes of autism involve a combination of genetic and environmental factors, such as oxidative stress, parental age, fetal infections, and fetal testosterone levels. Nutritional deficiencies, often related to selective eating behaviors in individuals with autism, also play a significant role in the condition [131],[132]. ASD encompasses a group of neurodevelopmental disorders characterized by impaired social communication, restricted behavior patterns, anxiety, and cognitive challenges. Growing evidence suggests a connection between the gut microbiota and the severity of ASD. Notably, the use of the antibiotic vancomycin has shown improvements in the severity and behavioral symptoms of ASD, indicating a potential role of the gut bacteria in ASD-related behavioral disturbances [133],[134]. Research on the role of microbiota in the pathogenesis of human ASD is partial and often inconsistent. However, there are exceptions, particularly in terms of the differences in specific bacteria such as *Prevotella*, *Firmicutes*, *Clostridiales*, *Clostridium perfringens*, and certain *Bifidobacterium* species [135]. Colonizing germ-free (GF) mice with fecal microbiota from individuals with ASD was described to induce ASD-like behaviors in the mice [136]. Children with ASD experiencing GI problems exhibit significantly higher levels of toxin-producing *Clostridium* in their gut compared with children without ASD and GI symptoms [137]. In ASD, the characteristic neurodevelopmental deficits often co-occur with GI symptoms like abdominal pain, diarrhea, and flatulence [138]. Changes in the gut microbiota's composition and metabolism have been observed in children with ASD and a mouse model of ASD [139]. Recent studies have shown that disruptions in the gut microbiota, which are crucial for brain development and function, may contribute to ASD-related behavioral deficits [140].

### 6.2. Multiple sclerosis

Multiple sclerosis (MS) is a global autoimmune and neurodegenerative disease affecting over two million people. It is characterized by neuroinflammation, lymphocyte infiltration into the CNS, demyelination, and axonal loss. Clinical symptoms include ataxia, coordination loss, hyperreflexia, spasticity, visual and sensory impairment, fatigue, and cognitive difficulties. Most patients experience a relapsing–remitting form of the disease, marked by recurrent symptom relapses and increasing neurological decline over time [141]. MS is an inflammatory disease marked by immune-mediated demyelination of the neural axons, leading to a range of neurological disorders, including motor, sensory, visual, autonomic, and cognitive impairments [142]–[144]. MS is marked by cognitive impairment, dyskinesia, muscle spasticity, numbness, fatigue, depression, sexual dysfunction, anxiety, vision loss, dizziness, and GI dysfunction [145]. The pathogenesis of MS is believed to stem from the immune system, influenced by genetic and environmental factors [146]. Environmental factors, particularly microbes and their secreted products, play a critical role in the pathogenesis of MS [147]–[149]. Jangi, et al. [150] reported that MS patients exhibit increased levels of *Methanobrevibacter* and *Akkermansia*, along with decreases in *Butyricimonas*. These changes correlate with variations in the expression of genes related to dendritic cell maturation, interferon signaling, and nuclear factor-kappa B signaling pathways in circulating T cells and monocytes. Patients on disease-modifying therapy (DMT) show increased abundances of *Prevotella* and *Sutterella* and decreased *Sarcina* compared with untreated patients.

### 6.3. Alzheimer's disease

Alzheimer's disease (AD) is a neurodegenerative disorder that leads to a weakening of activities, memory, thinking, language, and cognitive abilities, collectively referred to as dementia in older adults. The overproduction and deposition of amyloid-beta (Aβ) peptides and the translocation of microbes and their products into the brain can trigger neuroinflammation and neurodegenerative changes in AD. Thus, AD is often connected with an increasing accumulation of cerebral Aβ [150],[151]. As the most common form of dementia, Alzheimer's disease is a progressive neurodegenerative condition marked by the buildup of Aβ peptides in the brain [152]. The risk factors for Alzheimer's disease include age, gender, head injuries, cardiovascular diseases, lifestyle, environmental factors, diet, infections, genetic factors, obesity, and other conditions such as diabetes [153]. The disease has also been related to increased inflammation, and inflammatory cytokines can contribute to improved aggregation of Aβ and tau phosphorylation. These processes can lead to neurotoxicity and neurodegeneration as a result of neuroinflammation [154]–[156]. Aβ, a 40–42 amino acid peptide derived from the proteolytic cleavage of amyloid precursor protein (APP), plays a significant role in AD's pathogenesis by triggering neuroinflammatory responses in AD [157]. Previous research has suggested that the pathogenesis of AD is linked to peripheral infections that can lead to neuroinflammation in the CNS [158],[159]. Moreover, earlier studies have established potential mechanistic connections between AD pathology and various types of infections, including spirochete, fungal, and *Chlamydia pneumoniae* infections [160],[161]. One study showed that *H. pylori* increases tau hyperphosphorylation, a process attenuated by glycogen synthase kinase-3β (GSK-3β). This pathway has been connected to outer membrane vesicle-stimulated tau hyperphosphorylation and cognitive impairment [162]. Elevated Aβ deposition of proinflammatory mediators through the microglia, including iNOS, ROS, COX2, and NF-κB, contributes to neuroinflammation in AD's pathogenesis [163]. AD patients show alterations in their stool microbial profile, including decreased Firmicutes and *Actinobacteria* counts and increased Bacteroidetes compared with controls. Within Firmicutes, families like *Ruminococcaceae*, *Turicibacteraceae*, and *Clostridiaceae* are less abundant in AD patients [164]. Changes in the gut microbiota can activate proinflammatory cytokines and increase gut permeability, potentially leading to the translocation of Aβ oligomers from the intestine to the brain. Vogt, et al. [165] injected Aβ 1–42 oligomers into the gastric wall of mice and observed that it contributed to neuroinflammation and AD [166]. The inflammasome and its products have also been implicated in AD's pathogenesis, with higher expression of IL-1b and IL-18 reported in the microglia, astrocytes, and neurons surrounding Aβ plaques or in the plasma of AD patients [167]–[171].

### 6.4. Parkinson's disease

Parkinson's disease (PD) is a neurodegenerative state characterized by impaired motor abilities due to dysfunction in the dopaminergic nigrostriatal system [172]. PD exhibits a range of symptoms, including motor issues like slowness of movement, rigidity, and resting tremors. Additionally, it presents with nonmotor symptoms such as cognitive disturbances, depression, mood changes, sensory alterations, sleep disturbances, and autonomic dysfunctions, contributing to significant disability. This broad clinical spectrum is connected with accumulation in the central and peripheral nervous systems. [173],[174]. Parkinson's disease has a multifactorial etiology, likely arising from the interplay of environmental and genetic factors. Major contributors include toxic chemical exposure, head injuries, ecological elements, genetic and epigenetic risk factors, and the natural process of aging [175],[176]. Like many diseases, PD patients exhibit a distinct microbiota composition compared with healthy controls or individuals with other neurological disorders [177]–[179]. In seeking to understand PD, gene–environment interactions are crucial. Genome-wide association studies have identified genetic variants associated with PD, including mutations in at least seven genes (*LRRK2*, *ATP13A2*, *PINK1*, *DJ-1*, *SNCA*, *VPS35*, and *PARK2*) [180]. In early-onset PD patients with mutations in the *PARK2* and *PARK2* genes, and in mice lacking the mitochondrial serine/threonine protein kinase PINK1, an exacerbated NLRP3 inflammasome response was observed in the microglia and macrophages [181]. Emerging evidence suggests that α-synuclein pathology begins in the ENS before spreading to the CNS during the early stages of the disease, accompanied by specific digestive symptoms [182]–[184]. Notably, TLR4 is reported to interact with misfolded α-synuclein, leading to downstream microglial responses, the production of proinflammatory cytokines, and the promotion of oxidative stress [117]. Additionally, fecal samples and sigmoid mucosal biopsies from PD patients showed a higher abundance of putative proinflammatory bacteria and a reduction in bacteria with anti-inflammatory properties. This corresponds to the inflammation-related misfolding of α-synuclein and the pathology of PD in the CNS [185]. In this context, microbial-directed therapies, such as probiotics, are emerging as potential treatment options. Probiotic supplementation is believed to offer various health benefits through the diverse functions of live microorganisms, including inhibiting pathogen colonization, modulating and normalizing the microbiome and its function, exerting immunomodulatory effects (e.g., reducing inflammation), and improving the host's epithelial barrier function. Remarkably, several studies using PD animal models have demonstrated the potential neuroprotective effects of probiotics in reducing dopaminergic neuronal degeneration [179].

## 7. Probiotic therapy and possible mode of action in the gut–brain axis

**Figure 3.**
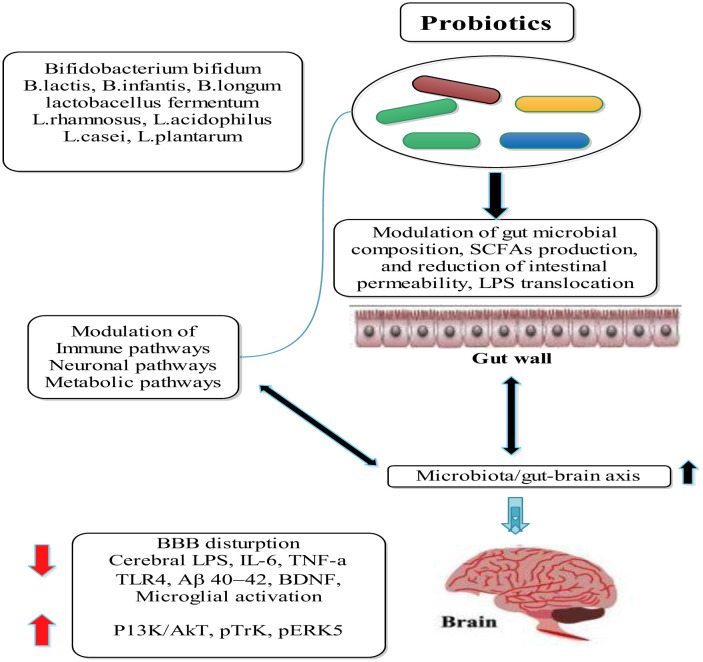
Outcome of probiotics on the modulation of the gut–brain axis. Specific strains such as *B. bifidum*, *L. rhamnosus*, and *Lactiplantibacillus plantarum* improve gut microbial composition, enhance SCFA production, and reduce gut permeability and systemic inflammation. These actions support immune, neuronal, and metabolic balance, ultimately contributing to brain health (↑ and ↓ indicate up- and down-regulation of molecular pathways, respectively). Adapted and slightly modified from reference [225].

Probiotics are considered a safe alternative treatment for various diseases and disorders. According to the United States Food and Drug Administration (FDA), probiotics are live microbes that, once administered in suitable amounts, deliver a health benefit to the host. Probiotics can be reduced into groups such as probiotic foods, direct microbial feeds, probiotic drugs, and genetically modified or designer probiotics [186]. The effect of probiotics on the development of the microbiota/gut–brain axis is explained in Figure 3. Certain dietary styles, like the Mediterranean diet, which is rich in plant-based foods and fiber, promote the growth of beneficial bacteria [187]–[189]. Some dietary supplements, such as omega-3 fatty acids, are used in the treatment of depressive disorders [190], though most dietary supplements still lack scientific evidence [191]. Probiotics typically consist of bacteria, including *Lactobacillus*, *Bifidobacterium*, and *Bacillus*, with a few strains of yeast, such as *Saccharomyces*, also included in probiotic cultures. According to the consensus panel of the International Scientific Association for Probiotics and Prebiotics, probiotics' benefits are attributed to specific strains within certain classes of bacteria, such as *Lacticaseibacillus casei* and *Bifidobacterium bifidum* [192]. Probiotics that, when consumed in appropriate quantities, yield positive psychiatric effects on psychopathology and provide CNS-related health benefits, are often referred to as “psychobiotics” [193]–[195]. Moreover, probiotics have been shown to decrease the expression of receptors for the repressive neurotransmitter GABA and the expression of cFos, a marker of neuronal action in the brain [196],[197], possibly by modulating the gut–brain axis [198]. Probiotic interventions for neurodegenerative diseases, such as AD, have recently been developed. In a double-blind, randomized, placebo-controlled trial involving 30 AD patients, a probiotic containing *Lactobacillus acidophilus*, *L. casei*, *B. bifidum*, and *Limosilactobacillus fermentum* was administered for 12 weeks [199]. Probiotics offer therapeutic potential through their modulation of various signaling pathways involved in inflammation, anti-tumor activity, antioxidant processes, and more. In particular, they affect TNF-α activation pathways. For instance, *Lactobacillus lactis* ML2018 can inhibit pathways such as NFκB and MAPK, preventing the overproduction of proinflammatory factors and improving intestinal health [200]. To enhance mechanistic depth, one study integrated psychobiotics' modes of action, including the modulation of neuroinflammatory pathways (IL-6, TNF-α), neurotransmitter synthesis (GABA, serotonin), and vagus nerve-mediated signaling. The review categorized these mechanisms by probiotic strain and neurological disorder, offering a more detailed understanding of how gut microbes influence brain function. This refined approach provides a mechanistically grounded perspective that advances beyond general associations, supporting the therapeutic potential of psychobiotics [194]. Probiotic organisms can alter the gut microbiome's composition and function, impacting digestion, immunity, and CNS health [201],[202]. They also upregulate anti-inflammatory factors and downregulate proinflammatory cytokines, potentially reducing intestinal inflammation [203]. Probiotics modulate various host immune functions, including innate and adaptive immunity [204], and they enhance phagocytosis and boost antibody secretion, leading to improved pathogen defense [204]. The bidirectional communication among the brain, intestines, and intestinal microbiota, known as the brain–gut axis, primarily arises through the autonomic nervous system (ANS), particularly the vagus nerve. Research indicates that 90% of the signals in this axis travel from the intestines to the brain; only 10% move in the opposite direction. Vagotomy, the cutting of the vagus nerve, does not disrupt intestinal function, highlighting the importance of the brain–gut connection [205]. The vagus nerve serves as the link between the intestine and the brain, allowing microorganisms to influence neuroinflammation. Manipulating this connection, such as translocating the vagus nerve, can reduce the incidence of AD and prevent microbial proliferation. Cognitive impairment in AD is associated with intestinal dysfunction and can potentially be managed by probiotics to improve bowel motility. Probiotics are beneficial microorganisms that can enhance the gut microbiota in AD patients [206]. Probiotics have demonstrated neuroprotective effects in mouse models of 1-methyl-4-phenyl-1,2,3,6-tetrahydropyridine (MPTP)- and rotenone toxin-induced PD [207]. Supplementation with a probiotic cocktail containing *L. rhamnosus* GG, *Bifidobacterium animalis lactis*, and *L. acidophilus* improved butyrate production, rescuing nigral dopaminergic neurons from MPTP- and rotenone-induced neurotoxicity. This effect was associated with the elevated brain-derived neurotrophic factor (BDNF) and glial cell line-derived neurotrophic factor (GDNF), helping cell survival and proliferation and the inhibition of monoamine oxidase B (MAO-B), enhancing dopamine synthesis and dopaminergic neuron survival. In a 6-hydroxydopamine (6-OHDA) mouse model, a probiotic mixture of nine bacterial strains conferred neuroprotection by reducing nigral dopaminergic neuronal loss. This protection was mediated through the activation of peroxisome proliferator-activated receptor gamma (PPAR-γ) by microbial byproducts, leading to anti-inflammatory and antioxidant activities, along with an upregulation of BDNF and its prosurvival pathways [208]. Preclinical studies and human trials indicate that probiotics, especially psychobiotics, offer promise for neural disorder diseases (NDDs) and brain health. However, several factors, including the specific strains, probiotic dosage, treatment duration, and the precise molecular mechanisms, require further investigation. Limitations include individual variations (genetics, environment, diet, gender) and the small sample sizes in clinical studies [209]. Studies involving healthy individuals show that supplementation with *Bifidobacterium* and *Lactobacillus* strains, namely *Lactobacillus helveticus* and *B. longum*, can reduce anxiety, depression, and stress-related behaviors [210]. In a follow-up study, probiotic supplementation was associated with reduced cortisol, anxiety, and depression levels, even in human subjects with low stress and cortisol levels [211],[212]. Liang, et al. [213] demonstrated that treating rats with *L. helveticus* NS8 improved chronic stress-induced depression and cognitive dysfunction. In a human study, a combination of *L. helveticus* R0052, *Bifidobacterium longum* R0175, and *Lactiplantibacillus plantarum* R1012 showed positive effects on anxiety- and depressive-like behavior, as previously observed in mice [214]. Bravo, et al. [197] reported that chronic treatment of healthy mice with *Lacticaseibacillus rhamnosus* reduced anxiety-related behaviors and induced region-dependent changes in transcripts encoding the metabotropic γ-aminobutyric acid receptor GABAB1 b, with a mouse strain effect observed in a follow-up study [215]. In a clinical trial with PD subjects, probiotic supplementation downregulated the expression of proinflammatory cytokines such as interleukin-1 (IL-1), IL-8, and TNF-α, while upregulating transforming growth factor beta (TGF-β) and peroxisome proliferator-activated receptor gamma (PPAR-γ) in the peripheral blood mononuclear cells (PBMCs) compared with a placebo [216]. A separate study of 60 PD patients reported that a multistrain probiotic supplement increased fatty acid levels in the brain, which are crucial for brain function, learning, memory, and neurogenesis. This effect was observed in a clinical trial with healthy subjects as well, in which a multistrain probiotic was shown to reduce activity in a brain network associated with the insula, a region involved in mood regulation and decreased attention to negative emotional stimuli [217]. Probiotic supplementation has shown therapeutic potential in multiple sclerosis (MS) and major depressive disorder (MDD). In MS, it has improved disability status, mental health, and various inflammatory and metabolic parameters [218],[219]. For MDD patients, the administration of probiotics, such as *L. acidophilus*, *L. casei*, and *B. bifidum*, significantly reduced depressive symptoms compared with a placebo [220]. Limited studies on probiotics for PD are available. One study reported that PD patients with chronic constipation experienced improvements in fecal consistency, bloating, and abdominal pain after taking fermented milk containing *L. casei* Shirota for five weeks [221]. Probiotics may have the possibility to counteract alterations in PD patients' microbiota composition, improve GI function, reduce gut leakiness, and decrease inflammation in the ENS. These improvements in GI function might enhance the absorption of levodopa and mitigate motor and cognitive impairments, including anxiety, depression, and memory difficulties, which are common symptoms in PD patients [222]–[224]. In children with ASD, probiotic supplementation for three months (aged 5–9 years) led to improvements in GI microbiota, GI symptoms, and the severity of ASD symptoms, behaviors, and overall functioning [225].

There is growing interest in the potential benefits of probiotics for treating or preventing neurologic illnesses. The studies on probiotic therapy for neurological disorders and gut health and their outcomes are given in Table 1. However, to assign them a role in decreasing neurologic symptoms or reducing the occurrence of neurodevelopmental disorders, a more thorough understanding of their mechanisms of action is needed, and the microbiota–gut–brain axis has been the focus of numerous related studies. The etiopathogenesis of ASD and certain other neurologic infections will become clearer with further research into the effectiveness of probiotics in altering these links, and new potential targets for treatment could be discovered [226]–[229]. Ghezzi, et al. [226] studied the role of probiotics and diet in the management of neurological diseases, and their study revealed that the use of probiotics could reduce neurological disorders such as AD, PD, ASD, anxiety, depression, and stress. Probiotics were recommended to be considered as an adjunct therapy to manage metabolic and psychiatric diseases. *Bifidobacteria* and *Lactobacillus* strains are known to be powerful probiotics that support the gut–brain system's reciprocal interactions; however, extensive randomized clinical trials are suggested to understand their entire potential. In another study, a meta-analysis was carried out for the first time, collecting data on probiotic supplementation in patients with different neurological disorders. The results of this study showed that probiotic supplementation improved C-reactive protein (CRP), malondialdehyde (MDA), insulin, homeostatic model assessment for insulin resistance (HOMA-IR), triglycerides, very low-density lipoprotein (VLDL) cholesterol, and high-density lipoprotein (HDL) cholesterol levels but did not affect other metabolic parameters [230]. Probiotic effects in animal models and clinical trials in humans with neurological disorders were assessed by Deidda and Biazzo [228] reported that probiotic supplement treatments are valuable tools with beneficial future therapeutic avenues for neurological conditions; however, they also mentioned the fact that still more clinical data are needed to demonstrate that correcting the dysbiotic state would restore physiological brain functions.

**Table 1.** Clinical and preclinical studies evaluating psychobiotics in neurological and stress-related disorders.

Disease/condition	Model (animal/human)	Treatment/probiotic strain	Key outcomes	Ref
Alzheimer's disease	Rat (β-amyloid-induced model)	500 mg probiotic daily via gavage for 4 weeks before and 2 weeks after β-amyloid (1–42)	Improved learning capacity and long-term potentiation (LTP)	[230]
Autism spectrum disorder	Human clinical trial	*L.acidophilus* (twice daily for 2 months)	Reduced urinary D-arabinitol, a metabolite linked to *Candida* overgrowth	[231]
Intestinal cancer prevention, liver function	Adult human (gut–stomach)	*Bifidobacterium pseudocatenulatum*	Modulated gut–bone axis, reduced inflammation, blocked TLR4/NF-κB signaling	[232]
Parkinson's disease	Adult human	Fermented milk with multistrain probiotics and prebiotic fiber	Increased bowel movement frequency	[233]
Alzheimer's disease	Adult human	Multistrain probiotic: *L. acidophilus*, *L. casei*, *B. bifidum*, and *L. fermentum*	Improved MMSE scores and β-cell function; reduced CRP, insulin resistance, and malondialdehyde	
Autism spectrum disorder	Children (4–16 years)	*L. plantarum* WCSF1	Increased beneficial bacteria; improved stool consistency and behavior scores	[234]
Depression, anxiety, stress	Adult human (IBS patients)	*B.longum* NCC3001	Reduced depression Hospital Anxiety and Depression Scale (HAD scores); improved emotional reactivity to negative stimuli	
Stress	Healthy adults	*L. acidophilus* Rosell-52, *B. longum* Rosell-175	Reduced stress-induced GI symptoms; no major psychological effects	
Stress	Healthy adults	*L. helveticus* R0052 + *B. longum* R0175 (30-day placebo-controlled trial)	Decreased psychological distress and urinary free cortisol levels	[225]

Table 1 summarizes the key preclinical and clinical studies evaluating the efficacy of probiotic interventions in neurological disorders and stress-related conditions. Treatments range from specific bacterial strains to multistrain formulations, with outcomes including behavioral improvements, cognitive enhancement, immune modulation, and gastrointestinal symptom relief. The references relate to primary studies and represent diverse human and animal model investigations.

## 8. Administration of probiotics and response in the gut–brain axis and neuronal disorders

Administration of probiotics can positively influence the gut–brain axis, helping to restore proper intestinal metabolism and support cognitive functions. Specifically, the probiotics *B. bifidum* novaBBF7, *B. longum* nova BLG2 (at 5 mg/mL), and *Lacticaseibacillus paracasei* TJB (at 10 mg/mL) improved intestinal homeostasis, leading to enhanced brain activity and reduced neuronal cell loss. In addition, SCFAs produced by these probiotics, such as butyric acid, acted as signaling molecules, activating various mechanisms. Generally, one study suggested that maintaining a healthy intestinal microbiota using probiotics like *B. bifidum* novaBBF7, *B. longum* novaBLG2, and *L. paracasei* TJB8 can help preserve cognitive function [235]. The formulation consisted of food-associated strains and human gut bacteria, given at a dosage of 10^9^ CFU (colony-forming units) per mouse per day. The study used a mouse model of acute inflammation induced by a single intraperitoneal injection of lipopolysaccharide (LPS) at the end of the administration of probiotics. The results revealed that prolonged administration of the multistrain probiotic formulation had several beneficial effects. It prevented the LPS-induced elevation of proinflammatory cytokines in specific brain regions, such as the hippocampus and cortex, as well as in the gastrointestinal system. Additionally, the probiotic treatment led to a significant increase in hippocampal neurogenesis, generating new neurons in the hippocampus, a brain region that is crucial for learning and memory. Moreover, the administration of probiotic microflora was associated with enhanced gut barrier function, as indicated by the increased expression of epithelial junction proteins in the colon [236]. Another study investigated how probiotics influence the microbiota–gut–brain axis and cognitive function in aging animal models. The researchers focused on a probiotic blend called ProBiotic-4 containing *Bifidobacterium lactis*, *L. casei*, *B.bifidum*, and *L. acidophilus*. They tested its effects on senescence-accelerated mouse-prone 8 (SAMP8) mice, a model known for rapid aging and cognitive decline [237]. For 12 weeks, the SAMP8 mice received oral ProBiotic-4 supplementation. The treatment had several beneficial effects, including improved memory, reduced damage to neurons and synapses, and decreased brain inflammation. Additionally, it positively influenced the composition of the gut microbiota, strengthened the intestinal and blood–brain barriers, and lowered inflammation markers such as interleukin-6 and TNF-α. [238]. ProBiotic-4 also reduced harmful lipopolysaccharide (LPS) levels in the blood and brain, decreased the activation of inflammatory pathways (such as TLR4 signaling and nuclear factor-kappa B translocation), and lessened DNA damage and inflammatory responses [239].

Another study investigated the impact of the probiotic *L. rhamnosus* IMC 501 on zebrafish (*Danio rerio*) after 28 days of dietary administration. The research revealed differences in shoaling behavior and changes in the brain, including the expression levels of the brain-derived neurotrophic factor (BDNF) and genes associated with serotonin signaling and metabolism. The study also found alterations in the zebrafish microbiota, specifically an increase in Firmicutes and a trend towards reduced Proteobacteria. These results suggest that specific probiotics can influence the gut microbiota, brain function, and behavior in zebrafish, indicating the potential for manipulating the microbiota–gut–brain axis in this model organism [240]. That study explored the interactions between the environmental pollutant perfluorobutane sulfonate (PFBS) and probiotic microbes in zebrafish. The researchers exposed adult zebrafish to low and high concentrations of PFBS (10 mg/L and 100 mg/L) with or without dietary administration of probiotic bacteria. The study investigated connections along the gut–brain axis, specifically focusing on neurotransmission signaling, immune function, and the hypothalamic–pituitary–adrenal (HPA) axis. The study exposed dynamic interactions between PFBS and probiotic bacteria, with diverse patterns detected in terms of PFBS concentrations, sex, and toxic indices. Probiotic and PFBS interactions mainly influenced neural signal transduction and immune responses. These interactions had minor effects on neuronal integrity and the HPA axis. Probiotics may play a role in mitigating the neurotoxic effects of pollutants in aquatic environments. The study acknowledges that probiotic supplements in fish feed can be absorbed by zebrafish, but the exact quantity of probiotic bacteria uptake remains unclear. The presence of PFBS pollutants may affect the uptake rate of probiotic-supplemented feed, which requires further investigation. Future studies may also explore how probiotic *L. rhamnosus* shapes the gut microbiota and influences the bioaccumulation of PFBS, shedding light on whether variations in neurotoxicology result from direct or indirect interactions between PFBS and probiotics [241].

Another study investigated the impact of probiotic pretreatment with *L. helveticus* R0052 and *B. longum* R0175 on the apoptotic pathway in the hippocampus of a rat model exposed to LPS, an endotoxin often used to induce inflammation. The expression of the antiapoptotic protein Bcl-2 increased in the hippocampus of rats exposed to LPS and treated with probiotics. The probiotic pretreatment attenuated the activation of caspase-3, a protein involved in cell death, by upregulating procaspase-3 and downregulating cleaved caspase-3 [242]. The study examined different dosing schedules, administering ProBiotic-4 at varying frequencies 0, 1, 3, or 7 times per week—over four weeks. The most substantial effects were observed with the 3× per week oral dosing. This dosing regimen was selected for subsequent studies in gut–brain communication, suggesting its potential relevance to physiological parameters [243]. Abnormal processing of myloid precursor protein (APP) is associated with the generation of β-amyloid (Aβ) plaques, a hallmark of AD. The administration of the probiotic ethanolic precipitate significantly reduced the level of Aβ in a rat model. This reduction in Aβ is a positive outcome, as the accumulation of Aβ is implicated in the pathogenesis of AD. The administration of the probiotic ethanolic precipitate effectively improved scopolamine-induced amnesia in mice. Scopolamine is a drug that impairs memory and is often used in animal models of memory impairment. These results suggest that *L. helveticus* IDCC3801-mediated fermented milk may have the potential to improve the memory deficits associated with Alzheimer's disease by influencing amyloid precursor protein metabolism. That study provides insights into the potential cognitive benefits of probiotic interventions for AD-related memory issues [244]. Administration of *L. plantarum* MTCC1325 improved AD symptoms in rats [245]. A probiotic mixture containing various strains improved oxidative stress in AD mice. It affected redox enzymes, enhanced superoxide dismutase (SOD) activity, and reduced carbonyl and 4-hydroxy-2-nonenal levels. The study showed that SLAB51 could mitigate aging and AD-associated oxidative stress and damage through the Sirtuin-1 pathway [246]. A study with 25 AD patients who received probiotics daily for 12 weeks showed improved cognitive function, increased serum glutathione (GSH), and reduced levels of 8-OHdG in the serum, indicating potential benefits for AD patients. However, there was no notable effect on total antioxidant capacity [247]. In a randomized, double-blind, placebo-controlled clinical trial, probiotics containing specific strains were administered to PD patients for 12 weeks. The probiotics led to a reduction in Movement Disorder Society–Unified Parkinson's Disease Rating Scale MDS-UPDRS scores, a decrease in high-sensitivity C-reactive protein and malondialdehyde, and an increase in glutathione levels. These results suggest the potential benefits of probiotics in PD patients [248].

In a study by Pinto-Sanchez, et al. [244], the effects of *B. longum* NCC3001 on anxiety and depression in patients with IBS were evaluated. Patients who received the probiotic powder had a greater reduction in depression scores compared with those given a placebo. Functional magnetic resonance imaging (FMRI) analysis revealed that the probiotic reduced responses to negative emotional stimulation in various brain areas compared with the placebo. Probiotics may have an impact on mood through the gut–brain axis. One study of a mouse model used a probiotic treatment to assess its effects on depressive behaviors induced by constipation. The results showed that probiotic administration improved depressive behaviors and reduced neuronal cell injury in the hippocampal CA3 regions. Additionally, the probiotic treatment decreased malondialdehyde (MDA) levels and increased total superoxide dismutase (T-SOD) activity. It also activated the AKT signaling pathway, increased Bcl-2 levels, and decreased Bax and cleaved caspase-3 levels, thus inhibiting neural apoptosis [249]. Multiple studies support the therapeutic potential of psychobiotics in modulating the gut–brain axis, but significant variation exists in terms of the probiotic strains, disease targets, and clinical efficacy. For instance, *L. plantarum* WCSF1 [29],[34] improved the microbiota composition and behavioral outcomes in children with autism, whereas *B. longum* NCC3001 showed significant antidepressant effects in adults with IBS [217]. Remarkably, multistrain formulations in AD and PD patients yielded broader systemic benefits, including improved cognitive scores and metabolic profiles, suggesting possible synergistic effects. In contrast, single-strain interventions in healthy individuals exposed to stress produced limited psychological changes but alleviated GI symptoms. Psychobiotics are a specialized class of probiotics that exert beneficial effects on mental health by influencing the gut–brain axis, distinguishing them from general probiotics, which primarily support gut and immune health. Microorganisms promote a balanced gut microbiota; psychobiotics, such as *L. rhamnosus* JB-1 [214],[240] and *B. longum* 1714 [199],[212], specifically modulate neuroactive compounds like serotonin, GABA, and dopamine; regulate the HPA axis; and reduce inflammation to improve mood, anxiety, and cognitive function. In contrast, general probiotics are typically used to enhance digestion, prevent gastrointestinal infections, and support immune responses. The growing interest in psychobiotics reflects their potential as adjunct therapies for psychiatric and stress-related disorders.

**Figure 4.**
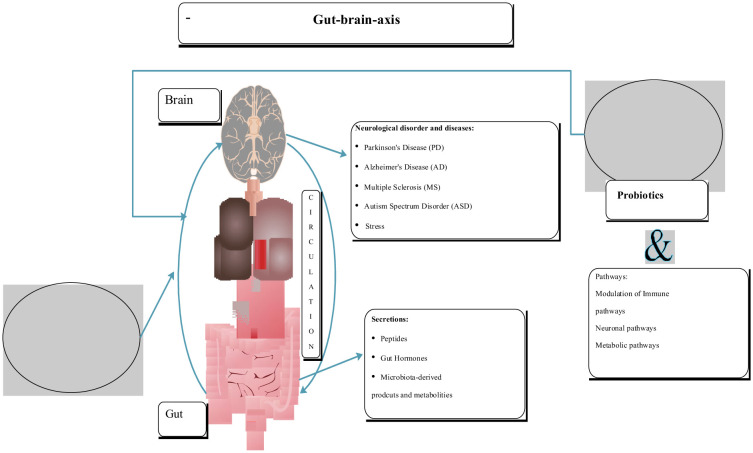
Microbiota and the gut–brain axis (pathway), neuroglial disorders, gut health, and probiotics and their effects.

A study by Mohammadi, et al. [246] investigated the effects of probiotic yogurt or capsules containing *L. acidophilus* LA5 and *B. lactis* BB12 on the mental health of petrochemical workers. The results indicated that a 6-week treatment with these probiotics improved mental health parameters as measured by a general health questionnaire and a depression, anxiety, and stress scale. In a study by Margret, et al. [248], a 2-week treatment with the probiotic formulation Probio'Stick (containing *B. longum* and *L. helveticus*) was found to attenuate the hypothalamic–pituitary–adrenal (HPA) axis and autonomic nervous system (ANS) activity in response to stress. This treatment also prevented the stress-induced reduction in neurogenesis in the hippocampus. The study suggested that probiotics can protect neuronal plasticity and maintain brain activity against stress-mediated brain circuitry insult by modulating the HPA axis. Another clinical trial focused on the oral administration of the probiotic VSL3 (a mixture of *Lactobacillus*, *Bifidobacterium*, and *Streptococcus* strains) in MS patients. The results indicated that VSL3 was associated with an anti-inflammatory peripheral innate immune response. This response was characterized by the reduced relative frequency of intermediate monocytes and decreased mean fluorescence intensity of human leukocyte antigen-antigen D related (HLA-DR) on myeloid-derived dendritic cells in MS patients (Figure 4).

## 9. Modulation of the gut–brain axis by fish and probiotics

Fish-derived bioactive compounds, particularly omega-3 polyunsaturated fatty acids (PUFAs), have been widely studied for their potential to modulate the gut microbiota and promote host health. Several *in vitro* and *in vivo* studies using mammalian models have demonstrated that dietary supplementation with omega-3 PUFAs can increase beneficial microbial populations such as *Lactobacillus* and *Bifidobacterium*, while reducing pro-inflammatory taxa like *Enterobacteriaceae*. However, it is important to note that some referenced studies within this context primarily use fish as model organisms to investigate the physiological and immunological responses to dietary inputs. While these studies provide valuable insight into host–microbiota interactions in aquatic species, further research specifically focusing on fish-derived PUFAs as direct treatments in mammals is warranted to clarify their translational relevance to human gut health [191], these compounds exhibit anti-inflammatory and antioxidant properties that support the gut–brain axis by promoting beneficial bacteria (*Lactobacillus* and *Bifidobacterium*), inhibiting pathogenic strains. Additionally, fish peptides enhance gut barrier function and influence neural pathways involved in stress and emotional regulation. Probiotics also contribute to gut–brain axis modulation. *Lactococcus lactis* WFLU12, for instance, improves neurotransmission and reduces stress in fish by upregulating neurotransmission-related genes and lowering stress markers [250]. Similarly, *Lactobacillus* species mitigate lead-induced neurotoxicity, regulate immune responses, and restore cognitive and behavioral functions disrupted by heavy metal exposure [251]. In zebrafish, *L. rhamnosus* IMC 501 affects behavior, brain-derived neurotrophic factor (BDNF) expression, and serotonin signaling, altering the gut microbiota's composition, specifically increasing Firmicutes and reducing Proteobacteria [252]. The neurotoxic effects of perfluorobutane sulfonate (PFBS) in zebrafish were also modulated by *L. rhamnosus*, which affects neurotransmitter profiles, acetylcholinesterase activity, and immune responses, with minimal impact on neuronal integrity [253]. Environmental toxins can also disrupt the gut–brain axis. Exposure to enrofloxacin induces anxiety-like behaviors in zebrafish by altering the microbiota's composition and affecting inflammation, neurotransmission, and stress-related biomarkers [239]. However, probiotic SLAB51 counteracts the toxic effects of bisphenol A (BPA) by restoring the gut microbiota's balance, improving liver health, and enhancing brain function. Co-administration of SLAB51 with BPA repairs the gut architecture, reduces harmful bacteria, and mitigates metabolic disruptions, including retinoid metabolism in females and anserine/glutamine levels in males, suggesting its role in detoxification [253]. Similarly, *Bifidobacterium* supplementation alleviates intestinal damage and metabolic dysbiosis caused by cyproconazole (CPZ) exposure in zebrafish by restoring acetate levels, promoting IL-22 secretion, and stimulating β-defensin production for gut repair [254]. Long-term supplementation with *L. rhamnosus* CECT8361 and *B. longum* CECT7347 influences zebrafish behavior, reducing anxiety-related bottom-dwelling tendencies after four months of treatment [255]. Finally, probiotics alter the gut microbiota's composition and influence gene transcription related to neurogenesis and stress in triploid Chinook salmon, but they do not fully mitigate the behavioral challenges associated with triploidy [256],[257]. The spectrum of antibiotic activity plays a pivotal role in shaping host-associated microbial ecosystems, with significant implications for health and disease outcomes. Narrow-spectrum antibiotics are designed to target specific bacterial taxa, either Gram-positive or Gram-negative organisms, thereby exerting minimal off-target effects on the commensal microbiota. This selective action helps preserve microbial diversity and maintain ecological stability within the gut, reducing the risk of antibiotic-induced dysbiosis [258]. In contrast, broad-spectrum antibiotics, such as fluoroquinolones, tetracyclines, and carbapenems, exhibit a comprehensive bactericidal effect, often resulting in the indiscriminate reduction of pathogenic and beneficial microbes [259]. This microbial perturbation can lead to substantial reductions in microbial alpha diversity, diminished production of key metabolites like SCFAs, and disruption of gut barrier integrity and mucosal immunity [260],[261]. In addition, wide-range antibiotic exposure has been associated with opportunistic infections, particularly *Clostridioides difficile*, and long-term infection of the gut–brain axis, with developing indications suggesting downstream effects on neuroimmune function and metabolic homeostasis [262],[263]. The ecological imbalance induced by such treatments may also contribute to the emergence of multidrug-resistant organisms, complicating therapeutic outcomes and public health [264]. Antibiotic treatment can disrupt the gut microbiota, reduce diversity, and cause a loss of beneficial taxa. Initial recovery may begin within 1–2 weeks, but full restoration of microbial composition and function can take several months to over a year, depending on factors such as antibiotic type, host age, and baseline microbiota [261]. Some microbial species may never fully recover, especially after repeated antibiotic courses [260]. Probiotics (e.g., *Lactobacillus*, *Bifidobacterium*, *Saccharomyces*) help re-establish microbial balance by outcompeting pathogens and modulating the host's immunity, reducing symptoms like antibiotic-associated diarrhea [193]. Prebiotics (e.g., inulin, FOS) selectively stimulate beneficial bacteria and support the recovery of short-chain fatty acid production [235].

## 10. Conclusions

The gut–brain axis is a complex, bidirectional communication system involving the gut microbiota that significantly affects physiological and neurological functions. This relationship is facilitated by various pathways involving the central and enteric nervous systems, in addition to microbial metabolites and immune signaling molecules. Probiotics, especially psychobiotics, have emerged as a promising treatment strategy to regulate this axis, potentially benefiting mental health and neurological conditions. Our review emphasizes the innovative use of psychobiotics to restore gut microbial balance, modulate immune responses, and promote the production of neuroprotective metabolites like short-chain fatty acids and vitamins. Moreover, bioactive compounds derived from fish provide new opportunities to support gut health and reduce neuroinflammation. Preclinical studies highlight the ability of probiotics to alleviate mood disorder symptoms, such as depression and anxiety, underscoring their role as complementary treatments in neuropsychiatric care. Despite these encouraging developments, additional research is needed to clarify strain-specific effects and the detailed mechanisms of action, and assess their long-term safety and efficacy in clinical environments. This work lays the groundwork for future translational studies focusing on developing targeted microbiota-based therapies, enhancing treatment options for neuropsychiatric and gut–brain axis-related conditions in humans and animals.

## Use of AI tools declaration

The authors declare they have not used artificial intelligence (AI) tools in the creation of this article.
